# The Herbal Compound Thymol Protects Mice From Lethal Infection by *Salmonella* Typhimurium

**DOI:** 10.3389/fmicb.2018.01022

**Published:** 2018-05-16

**Authors:** Yong Zhang, Yan Liu, Jiazhang Qiu, Zhao-Qing Luo, Xuming Deng

**Affiliations:** ^1^Key Laboratory of Zoonosis, Ministry of Education, Institute of Zoonosis, College of Veterinary Medicine, Jilin University, Changchun, China; ^2^Center of Infection and Immunity, The First Hospital, Jilin University, Changchun, China; ^3^Department of Biological Sciences, Purdue Institute for Inflammation, Immunology and Infectious Diseases and Purdue University, West Lafayette, IN, United States

**Keywords:** Type III secretion, Salmonella, anti-virulence, natural compounds, anti-inflammation

## Abstract

Type III secretion system (T3SS) is an essential pathogenic determinant for many important bacterial pathogens; it functions to thwart immune defense by delivering effectors into host cells. Because of its essential role in bacterial virulence, this machinery is an important target in the development of novel anti-virulence therapeutics. By using an effector-lactamase fusion reporter, we identified thymol, a monoterpene phenol derivative of cymene, as an effective inhibitor of the T3SS-1 of *Salmonella* Typhimurium. Our results indicate that thymol effectively protected mice against *S*. Typhimurium-induced mortality and pathological damages, suggesting that this compound can be developed for the control of infections caused by *Salmonella* species.

## Introduction

*Salmonella enterica* serovar Typhimurium (*S*. Typhimurium) causes gastrointestinal diseases in a wide range of hosts, including humans; these diseases impose an enormous economic impact on the society (MacFadden et al., 2016). For example, this pathogen greatly impacts the practice and safety of the poultry industry, a problem that has been further complicated by the emergence of strains resistant to multiple antibiotics (Chousalkar and Gole, 2016). Therefore, there is an urgent need for solutions to mitigate these challenges. Among the many factors important for *S*. Typhimurium virulence, the two type III secretion systems coded for by the pathogenic island SPI-1 and SPI-2, respectively, are essential for establishing systemic infections in hosts. SPI-1 codes for an injectisome and effectors that function mainly to induce the rearrangement of host cell actin cytoskeleton to form membrane ruffling, thus allowing the entry of the bacterium into nonphagocytic cells (Galán, 2001). This machinery and its effectors are required for the survival and replication of *S*. Typhimurium in macrophages, which is essential for systematic infections (Galán, 2001; Fass and Groisman, 2009).

The fact that these T3SSs are essential for bacterial virulence makes them ideal targets for the development of nontraditional anti-infection agents. A few compounds have been reported to target these protein transporters. For example, salicylidene acylhydrazides are able to protect calves from enteritis caused by *S*. Typhimurium (Hudson et al., 2007), Felise et al identified 2-imino-5-arylidene thiazolidinone as an effective inhibitor against animal and plant bacterial pathogens (Felise et al., 2008). Medicinal herbs, including many used in traditional Chinese medicine (TCM) have long been used to treat infectious diseases but the active components in these herbal medicines mostly remain elusive. A recent study showed that baicalin and related compounds target T3SS-1 of *S*. Typhimurium (Tsou et al., 2016). By using a reporter system that monitors the activity of SPI-1 under infection condition, we initiated a project to identify compounds present in components of TCM capable of inhibiting T3SS-1 of *S*. Typhimurium. Here we found that the herbal compound thymol (2-isopropyl-5-methylphenol) strongly inhibited the translocation of SipA into HeLa cells by *S*. Typhimurium at concentrations that do not detectably affect bacterial growth in bacteriological media. Further studies reveal that thymol could inhibited Salmonella invasion into HeLa cells and protects mice from *S*. Typhimurium-induced death.

## Materials and methods

### Compound screening, protein translocation assay and flow cytometry analysis

HeLa cells (1.5 × 10^4^) suspended in DMEM medium containing 10% FBS were seeded in 96-cell plates and the plates were incubated for 12 h at 37°C with 5% CO_2_. Overnight cultures of *S*. Typhimurium strains expressing the SipA-lactamase fusion were diluted 1:20 in LB containing 300 mM NaCl, the candidate compound was added to the cultures at the indicated concentrations. After growth for an additional 4 h, the bacteria were added to HeLa cells at an MOI of 50. Plates were centrifuged at room temperature for 10 min at 1,000 rpm and then were incubated at 37°C for 20 min. After removing the culture medium, cells washed three times with PBS were covered with 100-μl PBS containing 20-μl 6xCCF4/AM (Life Technologies). The reaction was allowed to proceed for 90 min at room temperature prior to visual inspection with an Olympus IX-81 fluorescence microscope.

For flow cytometry analysis, cells seeded in 6-cell plates were infected and allowed to react with CCF4/AM as descried above. After 3x washing with PBS, cells were lifted from the wells by trypsinization and centrifuged for 5 min at 1,000 rpm. Pelleted cells were gently resuspended in 400-μl PBS and were sorted in a BD LSR II flow cytometer to quantitate cells that emit blue or green fluorescence.

### Cytotoxicity assays

HeLa cells seeded in 96-cell plates with a density of 1.2x10^4^ per well were incubated overnight at 37°C with 5% CO_2_. After washed three times, cells were treated with thymol at the testing concentrations. After incubation for 12 h, the LDH release was measured by the Cytotoxicity Detection Kit (Roche Mannheim Switzerland) following the manufacturer's instructions. The absorbance at 490 nm was measured using a microplate reader (Tanon Tecan plus), LDH released from cells lysed by a buffer included in the kit was set at 100%. The percentage of LDH release was calculated with the follow formula: LDH release (%) = (Experimental LDH release-Spontaneous LDH release)/(Total LDH release-Spontaneous LDH release) × 100.

### Bacterial invasion

HeLa cells were seeded in 24-cell plates or on glass coverslips at a density of 1.2 × 10^5^ per well and incubated for 12 h at 37°C in a CO_2_ incubator. Overnight cultures of the testing strains were diluted 1:20 in LB broth containing 300 mM NaCl; thymol was added at the indicated concentrations and cultures were placed in a 37°C shaker (200 rpm) for 4 h. The bacteria were added to HeLa cells at an MOI of 50. After centrifugation for 10 min at 1000 rpm, infected samples were incubated at 37°C for 50 min. The culture medium was replaced with fresh DMEM containing 100 μg/ml gentamicin for 1 h. After washed the plates three times with PBS, 0.02% saponin (Sigma) was added per well to lyse the cells and appropriate dilutions of the lysates were plated onto bacteriological medium to obtain the CFU of the testing bacterial strains.

### Animal experiments

All animal experiments were performed in accordance with an animal use protocol issued by Jilin University (Protocol number: 20160315009). BALB/c mice were obtained from the Experimental Animal Center of Jilin University. Water containing streptomycin (5 g/L) was provided *ad libitum* for three days to condition the mice for salmonella infection (Barthel et al., 2003). Drinking water as libitum was offered 6-h before Salmonella infection. For infection, Bacteria strains grown in LB containing 0.3 M NaCl were washed twice in sterilized PBS and used for orogastric infection of 6–8 weeks old female BALB/c mice using a ball-tipped feeding needle. For survival assays, appropriately 5 × 10^7^ of bacteria were applied to different groups of mice (*n* = 15). The survival rate of the mice was determined by monitoring daily survival rates for 10 days. For infections used to monitor body weight loss, each mouse (*n* = 15) was infected with appropriately 1 × 10^6^ of bacterial cells. Body weight of mice was monitored for 10 days. In each case, control groups received sterile PBS. Thymol treatment were performed using the following regimen: three doses were given to the animal at 50 mg/kg of body weight by oral gavage one day before infection and then at 8-h intervals for another 5 days postinfection. For its effects in DSS-induced body weight loss, thymol was given to mice (*n* = 10 for each treatment) that had received 2.5% DSS for one week at 50 mg/kg of body weight for 5 days at 8-h intervals. The experiments were allowed to proceed for 15 days and the body weight was measured daily.

For histopathological study, mice were sacrificed by cervical dislocation at day 5 post-infection. To evaluate bacteria loads in spleens, livers and ceca, tissue samples were homogenized in cold PBS and serial dilutions of the homogenates were plated on LB plates under streptomycin selection, followed by overnight incubation at 37°C. For histological analysis, segments of the cecum, liver and spleen were fixed and embedded in paraffin according to standard procedures (Tournier et al., 1987). Cryosections were mounted on glass slides and stained with hematoxylin and eosin (H&E) (Cardiff et al., 2014). Pathological evaluation was performed independently by two pathologists. Cytokine levels were measured using an enzyme-linked immunosorbent assay (ELISA) according to the manufacturer's instructions (BioLegend).

## Results

### Thymol inhibits the translocation of a SipA-lactamase fusion by salmonella T3SS-1

**Figure 1:**
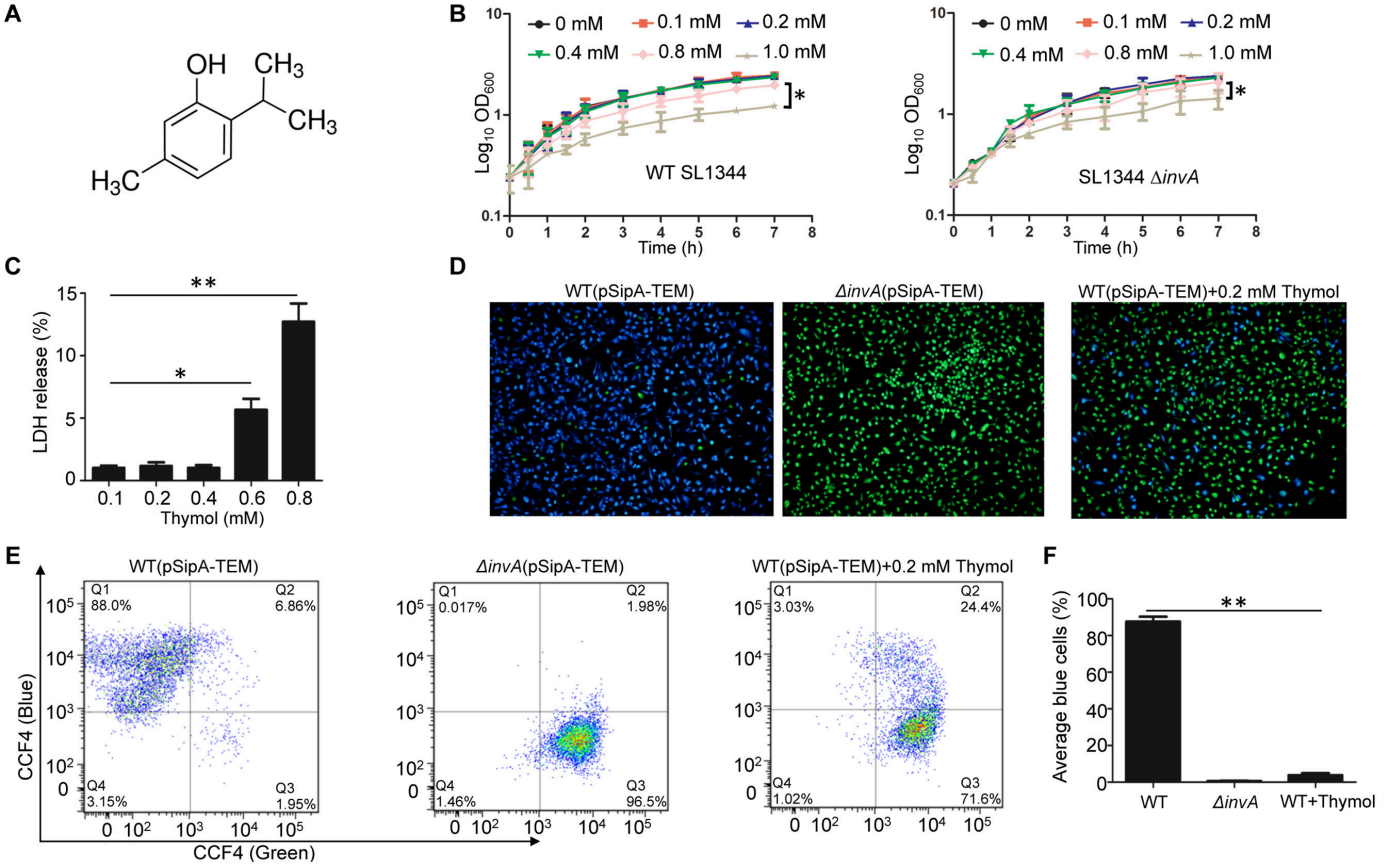
Thymol inhibits the translocation of a SipA-lactamase fusion by the T3SS-1 of *S*. Typhimurium. **(A)** The chemical structure of thymol. **(B)** The effects of thymol on the growth of *S*. Typhimurium. Thymol was added to bacterial cultures in LB broth at the indicated concentrations and the growth of the wild type (left) or the *invA* mutant defective in T3SS-1 bacteria was monitored by measuring OD_600_ at the indicated time points. Note that 0.4 mM thymol did not detectably affect bacterial growth. Results were from three independent experiments done in triplicate. Bar, s.e.m. (*n* = 3) ^*^p < 0.05. **(C)** Toxicity of thymol to mammalian cells. Thymol was added to HeLa cells at the indicated concentrations and the release of LDH was measured. Results shown were from three independent experiments done in triplicates. Bar, s.e.m. (*n* = 3) ^*^p < 0.05. **(D)** Representative images of the translocation of the SipA-lactamase fusion into HeLa cells. Wild type or the *invA* mutant of *S*. Typhimurium expressing the fusion was used to infect HeLa cells; thymol was added to the bacterial cultures for 4 h before being used for infection. Protein translocation was detected using the CCF4/AM substrate. Images were acquired with a fluorescence microscope. Blue and green cells represent positive and negative protein translocation, respectively. Note that thymol treatment reduced the number of blue cells. **(E)**. Quantitative analysis of the translocation efficiency by flow cytometry. HeLa cells infected with the indicated bacterial strains were loaded with the lactamase substrate CCF4/AM prior to being sorted by a fluorescence activated cell sorter. Note the significant reduction in cells emitting blue fluorescence upon thymol treatment. **(F)** The average of cells emitting blue fluorescence signals obtained in three independent experiments. Bar, s.e.m. (*n* = 3) ^**^p < 0.01.

To identify natural compounds capable of inhibiting SPI-1-mediated secretion into epithelial cells, we fused the TEM-1 β-lactamase to the carboxyl end of the well characterized T3SS-1 effector SipA (Zhou et al., 1999). Using this reporter system, we performed a screening and found that thymol, a phenol obtained from thyme oil or other volatile oils (Figure 1A), was able to significantly inhibit the translocation of SipA.

Thymol has been reported to have antimicrobial against *S*. Typhimurium, but the MIC reported by different studies varied greatly, ranging from 150 μg/ml (1.0 mM) (Olasupo et al., 2003) to 750 μg/ml (5.0 mM) (Chauhan and Kang, 2014). Nevertheless, consistent with these earlier reports, we found that 0.4 mM of thymol, which is less than half of the lowest reported MOI, did not detectably affect the growth of *S*. Typhimurium strain SL1344 in bacteriological media (Figure 1B). Meanwhile a mutant defective in SPI-1 grew indistinguishably to the wild type in media containing thymol at all of the tested concentrations (Figure 1B).

We also examined the cytotoxicity of thymol by measuring the cytoplasmic enzyme lactate dehydrolase (LDH) release from HeLa cells. 0.4 mM of thymol did not detectably cause damage to the membranes of HeLa cells (Figure 1C). To prevent the potential complications caused by high concentrations of thymol, we used 0.2 mM or less in our subsequent experiments.

Flow cytometry analysis indicated that close to 90% of the cells in samples infected with the wild type strain SL1344 received the SipA::TEM-1 fusion injected by T3SS-1. Treatment with 0.2 mM thymol reduced the translocation efficiency to about 20% (Figures 1D–F). As expected, few cells infected with the T3SS-1-defective mutant Δ*invA* expressing the same fusion emitted blue fluorescence signals (Figures 1D–F). These results indicate that thymol is capable of blocking the activity of T3SS-1 at concentrations that did not affect either bacterial viability or the integrity of mammalian cell membranes.

### Thymol protects mice from *S*. typhimurium-induced death

**Figure 2:**
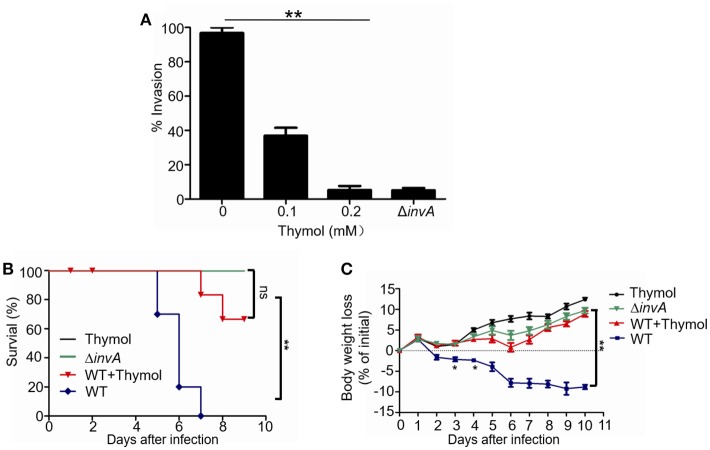
The protection of cells and mice from *S*. Typhimurium infection by thymol **(A)** The effects of thymol on the invasion of HeLa cells by *S*. Typhimurium. The indicated bacterial strains were used to infect HeLa cells. The rates of invasion were calculated by setting the values of internalized wild type bacteria in untreated samples as 100%. Bar, s.e.m. (*n* = 3), ^**^p < 0.01. **(B)** Thymol protected mice from body weight loss caused by *S*. Typhimurium infection. The body weight of groups of mice infected with the indicated bacterial strains was monitored daily. Note that mice infected with wild type bacterial exhibited body weight loss, which can be reversed by thymol. **(C)** Bacterial strains grown in LB were washed twice in sterilized PBS and used for orogastric infection of 6–8 weeks BALB/c mice using a ball-tipped feeding needle. For survival assays 5 × 10^7^ CFU bacteria of each strain were applied to different groups of mice (*n* = 10/strain), and the survival rate of the mice was determined by monitoring the survival daily for 10 days. Similar results were obtained in three independent experiments, and data shown are from one representative experiment done in triplicate. ^**^p < 0.01; ^*^p < 0.05. Statistic analyses were performed by Log-Rank test.

We next examined the effects of thymol on bacterial invasion into HeLa cells by the gentamicin protection assay (Collazo and Galán, 1997). Culturing the bacteria with 0.1 mM thymol for 4 h prior to infection reduced the rates of invasion to less than 10%, which was similar to those observed with the mutant defective in *invA*, a gene that is essential for bacterium-induced internalization (Ginocchio and Galán, 1995; Figure 2A). Thus, consistent with its ability to inhibit T3SS-1, thymol interferes with bacterium-induced entry into epithelial cells.

The strong inhibitory effects of thymol on the invasion of *S*. Typhimurium in cultured cell infections prompted us to evaluate its usefulness in protecting animals from infection caused by this pathogen. Mice pre-treated with streptomycin (Barthel et al., 2003) were infected with wild type or the Δ*invA* mutant. We first examined the loss of body weight of animals infected by low doses (appropriately 1 × 10^6^/mouse) bacteria (Li et al., 2015). Animals infected with wild type bacteria without thymol treatment exhibited steady body weight loss throughout the 10-day experimental duration, which can be effectively reversed by thymol (Figure 2C). As expected, animals infected with the Δ*invA* mutant or uninfected groups receiving thymol gained body weight as the experiments proceeded (Figure 2C).

In experiments aiming at determining the survival of the infected animals after being challenged by high doses (appropriately 5 × 10^7^/mouse) bacteria, no death was observed in groups infected with the Δ*invA* mutant in the entire 10-day experimental duration (Figure 2B). In contrast, more than 80% of the animals infected with the wild type bacteria died on the 6th day postinfection and all mice succumbed to the infection at day 7 (Figure 2B). Importantly, if mice were administered with thymol three times in 24 h at an 8-h interval at a dose of 50 mg/kg on the day prior to being challenged with wild type bacteria, followed by identical doses at 8-h intervals for 5 days, no death was observed at the 6th day postinfection (Figure 2B). At the 7th day, 80% of the animals treated with thymol survived and 70% of them survived to the 9th day postinfection (Figure 2B).

### Thymol alleviates the pathology induced *S*. typhimurium infection

**Figure 3:**
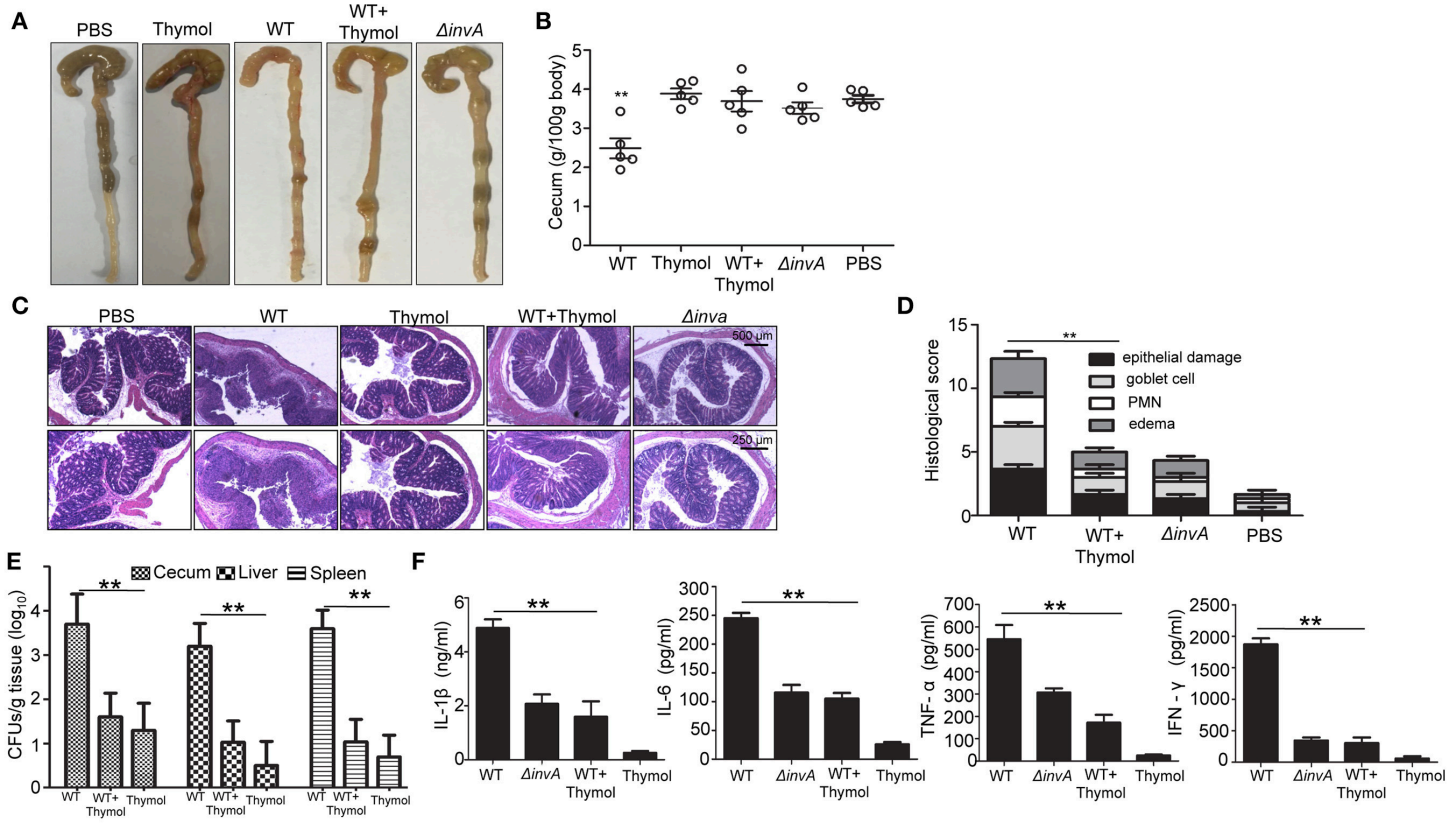
Thymol alleviates the pathology induced *S*. Typhimurium infection. **(A,B)** Morphological differences of the cecum. Streptomycin-treated mice were infected with wild type bacteria with or without thymol treatment or with the *invA* mutant. Cecum images **(A)** and weight **(B)** were acquired. Note that thymol treatment restored the morphology to controls received saline buffer or infected with the *invA* mutant. **(C,D)** Histopathology of the cecum. Sections of cecum from different groups of mice were stained with hematoxylin and eosin (H&E) to visualize epithelial cells and goblet cells **(C)**. The histopathological damage was scored **(D)**. Bar, s.e.m. (*n* = 3) ^**^p < 0.01. **(E)** Thymol reduces the bacterial load in several organs of the infected mice. The bacterial load in cecum, liver, and spleen were determined at 5 d after infection. Results shown were from one representative experiment done in triplicate. Similar results were obtained in three independent experiments. **(F)** The effects of thymol treatment in the production of several cytokines. Ceca of infected mice differently treated with thymol were measured for the indicated cytokines. All experiments were performed with tissues from at least three mice and similar results were obtained from at least three independent experiments. Bar, s.e.m. (*n* = 3); ^**^p < 0.01.

Consistent with earlier results that thymol did not affect mouse body weight gain (Figure 2C), ceca from mice treated with thymol displayed a morphology similar to those from control groups received PBS and those infected with the Δ*invA* mutant defective in the SPI-1 (Figure 3A). The ratio between cecum and body weight of these two groups of mice was also similar (Figure 3B). In contrast, this ratio was significantly lower in mice infected with wild type *S*. Typhimurium, which was restored in infected mice treated with thymol (Figure 3B). Histological examination after hematoxylin and eosin (H&E) staining showed that ceca from mice infected with wild type bacteria displayed submucosal edema, the loss of goblet cells, damage of the epithelial layer and the infiltration of polymorphonuclear granulocytes (PMN) in the lamina propria; again, these symptoms were alleviated when thymol was given to similarly infected mice (Figures 3C,D). Similarly, thymol treatment significantly reduced the amount of bacteria associated with such organs as spleen, liver and cecum, indicating reduced bacterial dissemination in thymol-treated mice (Figure 3E). We also measured the status of inflammation in these animals. The amounts of pro-inflammatory cytokines such as IL-6, IL-1β and TNF-α in the ceca were significantly higher in untreated animals infected with wild type bacteria, and thymol treatment significantly reduced the production of these cytokines (Figure 3F). Taken together, these results indicate that thymol can provide effective protection against *S*. Typhimurium infection in mice.

### The effects of thymol on DSS-induced body weight loss

**Figure 4:**
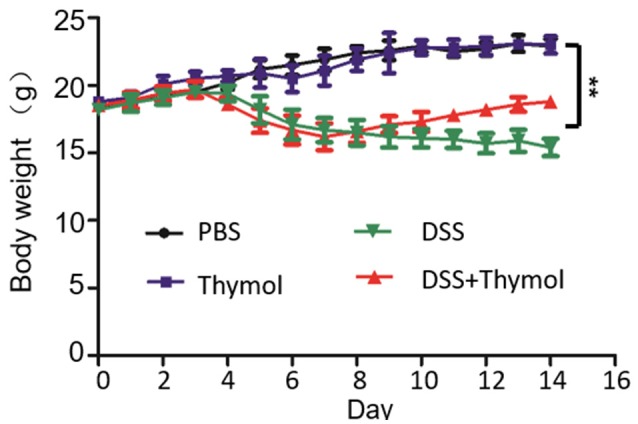
The effects of thymol on DSS-induced body weight loss. Thymol reversed body weight loss caused by the colitis-induced body weight loss in mice. Mouse groups (*n* = 10) were treated with the indicated agents as described in Materials and Methods. For treatment, thymol was given orally for 5 days at 8-h intervals after DSS had been administered for 7 days. Similar results were obtained in two independent experiments, and results shown were from one representative experiment. ^**^p < 0.01.

Thymol is known to have anti-inflammatory activity (Braga et al., 2006; Riella et al., 2012; Zhang et al., 2017); we thus determined its effects on body weight loss induced by dextran sulfate sodium (DSS), a compound that causes colitis in mice (Chassaing et al., 2014). Mice received PBS or thymol began to gain body weight 4 days after the experiments were initiated. When 2.5% DSS was included in drinking water for a week, body weight loss was apparent 4 days after receiving the compound and such loss continued throughout the 15-day experiment duration (Figure 4). After receiving DSS for a week, if the mice were given thymol at 50 mg/kg for 5 days at 8-h intervals, the body weight loss can be effectively reversed (Figure 4). These results indicated that thymol used at doses in our anti-infection treatment experiments had anti-inflammatory effects.

## Discussion

The emergence and wide spread of bacterial pathogens resistant to classic antibiotics pose a grave challenge to public health. One promising strategy to meet this challenge is to develop anti-virulence agents that do not indiscriminately kill bacteria, thus are less likely to induce resistance. The study of bacterial pathogenesis in the past three decades has identified many elements essential for bacteria to cause infection, which are ideal targets for anti-virulence therapeutics (Rasko and Sperandio, 2010). Among these, type III secretion systems widely used by Gram negative bacterial pathogens have been exploited for such purpose (Galán et al., 2014). Our identification of thymol, a natural monoterpene phenol present in many components of herbal medicine adds one agent to the list of compounds that inhibit the activity of the T3SSs (Duncan et al., 2012; Gu et al., 2015; Tsou et al., 2016).

Thymol has been shown to have antimicrobial activity at MICs ranging from 1 to 5 mM, probably due to the different bacterial strains and/or experimental conditions used. Under our experimental condition, we found that when used at as high as 0.4 mM, thymol obtained from Sigma did not detectably inhibit the growth of *S*. Typhimurium in broth, nor did it cause damage to the membranes of mammalian cells (Figures 1B,C). Furthermore, to ensure that the protective effects from its antimicrobial activity are minimal, in our experiments, we used 0.1 mM, which is less than 1/5 of the lowest reported MIC (Chauhan and Kang, 2014). Thus, the protection observed in our infection models should be mostly a result of attacking virulence factors associated with T3SS-1 and potentially T3SS-2. The effectiveness of thymol in treating infections was previously attributed to its antimicrobial activity (Essawi and Srour, 2000; Adams et al., 2009). Given the high level similarity among T3SSs from different pathogens, it is tempting to speculate that the inhibition of T3SS function contributed to the protection observed in these reports with pathogens that use T3SSs for virulence.

The inhibition of T3SS-1 by thymol can be achieved by multiple possible mechanisms. First, it can directly block the biogenesis of the translocation machinery by inhibiting gene expression. Second, it may cause malfunction of the machinery by direct interaction with and inactivating one or more of its essential components. Third, it may affect the expression and/or stability of one of more of its effectors.

Virulence-induced inflammation increases the fitness of *S*. Typhimurium in the gut by producing an electron acceptor that enable the pathogen to compete with fermenting gut microbes (Winter et al., 2010). Although inhibition of T3SS clearly is the primary factor for the protective effects of thymol, its potential impact on the host such as the inhibition of inflammation may also contribute to the observed protection.

A number of small molecules capable of inhibiting bacterial T3SSs have been reported (Duncan et al., 2012), including one that functions against T3SSs in diverse bacteria (Felise et al., 2008). Yet, the mechanisms of action for most of these agents remain elusive. Interestingly, the herbal compound bacailin and its derivatives inactivate T3SS effectors of *S*. Typhimurium by covalent binding (Tsou et al., 2016). It is also not clear whether bacailin is able to protect animals against *S. Typhimurium* infection. It will be interesting to determine whether combinations of these compounds can provide better protection than individual compound. In sum, the lack of antibiotic activity under working concentrations and the proven low toxicity make these compounds promising candidates for the development of novel anti-virulence agents.

## Author contributions

Z-QL and XD conceived the project; YZ, YL, Z-QL, and XD designed the experiments; YZ, YL, and JQ performed the research; YZ, YL, XD, and Z-QL wrote the paper and all authors made editorial input.

### Conflict of interest statement

The authors declare that the research was conducted in the absence of any commercial or financial relationships that could be construed as a potential conflict of interest.
